# Prospective cohort study of operative outcomes in laparoscopic cholecystectomy using operative difficulty grade-adjusted CUSUM analysis

**DOI:** 10.1093/bjs/znad046

**Published:** 2023-03-08

**Authors:** Isaac Tranter-Entwistle, Corin Simcock, Tim Eglinton, Saxon Connor

**Affiliations:** Department of Surgery, The University of Otago Medical School, Christchurch, New Zealand; Department of Surgery, The University of Otago Medical School, Christchurch, New Zealand; Department of Surgery, The University of Otago Medical School, Christchurch, New Zealand; Department of General Surgery, Christchurch Hospital, CDHB, Christchurch, New Zealand; Department of General Surgery, Christchurch Hospital, CDHB, Christchurch, New Zealand

The development of a learning health system requires a shift from intermittent retrospective review of outcomes to continuous data-driven understanding of surgical processes and patient outcomes. Doing so will require the routine prospective capture of high-quality structured data. This lack of structured data has been a significant impediment to the development of surgical data science^1,2^. Laparoscopic cholecystectomy, a high-volume procedure, represents an opportunity to address this, but application of these data will require a paradigm shift in outcome reporting.

As part of a systems-wide quality-improvement programme an end-to-end perioperative electronic workflow solution was refined and implemented at Christchurch Hospital, a tertiary referral centre, in 2013^3^. This system allows for end-to-end data capture, including wait listing, operation booking, synoptic operation note documentation, and prospective capture of surgical complications and outcomes. Coupled with this, Christchurch Hospital has undertaken a department-wide initiative to standardize processes in laparoscopic cholecystectomy to optimize outcomes and prevent bile duct injury (BDI)^4^. Integration of prospective data capture into the clinical workflow allows for the possibility of real-time outcome monitoring and quality improvement.

Cumulative summation analysis (CUSUM) is one such method for providing real-time reporting of outcome quality^5–9^. CUSUM is a sequential analysis technique that can be used to determine if performance deviation from a defined standard is significant. It is ideally suited to surgical data sets where there is a well-defined binary outcome, ever-increasing denominator, and well-established benchmarks for predefined outcomes. Retrospective application of this technique to the Bristol and Shipman cases suggests that use of this analysis technique would have raised concerns earlier^10^. Using a prospectively gathered data set of laparoscopic cholecystectomy operations, this paper aims to demonstrate how integrated electronic workflow solutions can facilitate perioperative data capture and real-time outcome monitoring.

Data were prospectively captured as part of clinical workflows at the time of patient presentation by the attending clinical team. Data from all adult patients presenting between 2015 and 2021 were included. Analysis was conducted in keeping with the TRIPOD checklist for prediction model development (*Appendix S1*). Documentation of operative process for laparoscopic cholecystectomy was standardized through the use of synoptic operative notes^3^. Synoptic note content was developed through focused consultant review before achieving departmental consensus with periodic review as part of continuous quality improvement by consultant consensus. This iterative process improvement resulted in a variable denominator of some data fields throughout the study period. Postoperative complications were entered at the time of occurrence by the clinical team, and date of death was imported automatically from a national central registry.

Risk-adjusted CUSUM analysis was conducted using a purpose-built Microsoft Excel spread sheet for Clavien–Dindo (CD) grade 3–5 complications (*Supplementary Methods*)^11–13^. CUSUM performance was also charted for major BDI. The low rate of BDI precluded the use of risk adjustment. To ensure the ongoing utility of quality monitoring resets were applied if the CUSUM score crossed a level of certainty with *P* < 0.0001, in keeping with the methodology described by Yap *et al*. and Spiegelhalter *et al*.^5,10^. For CD 3–5 complications risk adjustment was performed using the methodology described by Steiner *et al*.^11^. A logistic regression model using the first 2 years of data was created using the north shore operative difficulty grade split as straightforward (grade 1–2) and complicated (grade 3–4) to risk adjust for the significant impact operative difficulty grade has on patient outcomes^14,15^. The north shore scale was selected due to local departmental experience and literature showing its utility in understanding process^15,16^. Risk adjusted CUSUM (RA-CUSUM) charts were then constructed, setting the odds ratio to 0.66 for improvements and 1.5 for a deterioration in the complication rate^11–13,17–20^.

In total, 4663 patients underwent cholecystectomy during the study period. Synoptic operative note version 1 was used for 1908 (41 per cent) of these patients, version 2 for 621 (13 per cent) patients, and version 3 for 2134 (46 per cent) patients. Preoperative, intraoperative, and postoperative patient factors are detailed in *Tables S1* and *S2*. Overall, 100 (2 per cent) patients had CD 3–5 complications, 35 (0.75 per cent) had bile leaks, and two (0.04 per cent) had BDIs (*Fig. 1* and *Table S3*). RA-CUSUM analysis showed an acceptable rate of grade 3–5 complications over the study period (*Fig. 2*).

**Fig. 1:**
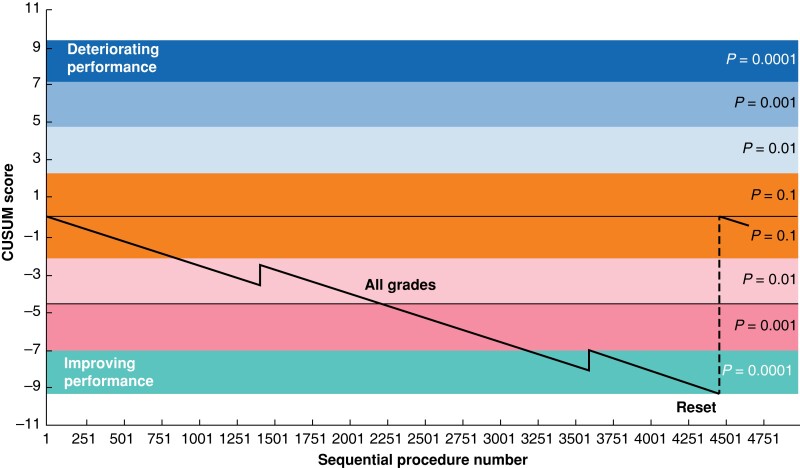
Cumulative summation analysis (CUSUM) for major bile duct injury (BDI) after laparoscopic cholecystectomy Acceptable failure rate set at 0.0015% and unacceptable at 0.004%.

**Fig. 2:**
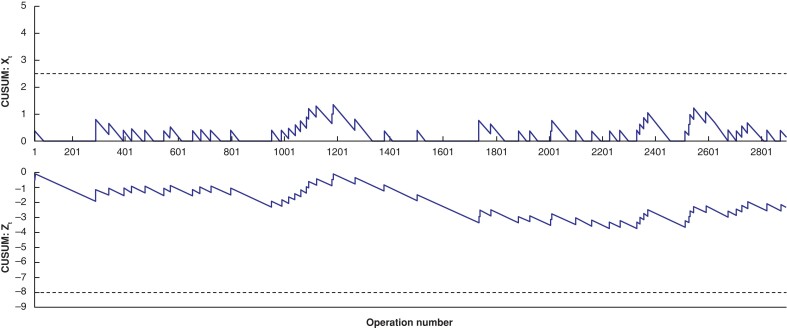
Risk-adjusted cumulative summation analysis (CUSUM) charts for Clavien–Dindo grade 3–5 complications

The study used CUSUM and RA-CUSUM methodology to demonstrate acceptable complication rates and a low rate of BDI throughout the study period (*Figs 1*, *2*). The utility of CUSUM analysis in monitoring surgical outcomes has previously been illustrated in colorectal and cardiothoracic operations^8,10^. In both instances, however, the analysis was performed as audit of retrospectively gathered data. Over the period of the present study the Christchurch general surgery department has undertaken a systematic effort to standardize the operative process and develop a culture of safety in laparoscopic cholecystectomy. This standard process, as described by Connor *et al*.^4^, includes cranial retraction of the gallbladder to 10 o’clock, identification of Rouviere’s sulcus or the posterior portal pedicle, and index dissection on the posterior leaf of peritoneum prior to achieving the critical view of safety. While it is not possible to say definitively if this quality-improvement process has led to the outcomes seen here, the prospective data capture allows for safety monitoring of processes in real time.

A shift from retrospective review and the presentation of annualized outcome data to real-time monitoring is needed. RA-CUSUM is one such method for doing so and allows both for departmental- and individual-level monitoring. One of the criticisms of this method is the need to account for case mix and difficulty, as more difficult operations lead to worse outcomes^8^. At a departmental level the large volume of operations should allow for robust comparison across centres. However, at an individual surgeon level the variation possible as a consequence of speciality, subspecialist interest, or practice norms means that case mix does need to be considered. At Christchurch Hospital there is a standard review process for any instance of common bile duct injury, including case review and process, as well as video analysis, if available. Focusing on the system rather than the individual prioritizes a culture of safety allowing for improvement rather than protectionism. For CD grade 3–5 complications, their higher incidence allows for risk stratification using operative difficulty grade. Operative difficulty grade is likely the most significant factor in predicting postoperative outcomes^21^, with Griffith *et al*.^21^ showing a reintervention rate of 20.4 per cent in grade 4 operations *versus* 7.6 per cent in grade 1 operations, falling to 3.6 per cent and 0.7 per cent, respectively, in a subspecialist practice. In this context, RA-CUSUM allows for safety monitoring of a complex system while undergoing iterative process improvements.

The process used in the development and capture of the data presented here illustrates the importance of integrating data capture into workflows using structured data fields. Christchurch Hospital has undertaken an iterative process of developing and integrating systems that allow for the prospective capture of structured data. As described by Sakowska *et al*.^3^, data capture through the use of scOPe is currently within the remit of many health systems. While structuring and capturing the data has been achieved, real-time feedback has not currently been actioned in practice. Currently, captured data are centralized for storage and management through a locally manged data warehouse. This system allows for the generation of real-time dashboards for clinical outcomes using Microsoft Power BI. Discussion at a departmental level has highlighted the acceptability and utility of integrating these data as part of real-time outcome monitoring into morbidity and mortality meetings. Significant drops in performance over time would then trigger focused reviews of practice and process. However, a paucity of data scientists and data-literate clinicians to build and maintain these dashboards, coupled with data-literate clinicians, has proven an impediment to progress. As surgical data science matures, the integration of ideas into practice will require not just the infrastructure, but also the surgical data scientists to implement and surgeons to utilise them.

## Supplementary Material

znad046_Supplementary_Data

## Data Availability

Data can be made available on reasonable request.
