# Supplementary material for: Prospective cohort study of operative outcomes in laparoscopic cholecystectomy using operative difficulty grade-adjusted CUSUM analysis
Source: Br J Surg. 2023 Mar 8;110(9):1068–71. doi: 10.1093/bjs/znad046 (PMC10416680; doi:10.1093/bjs/znad046)
Supplement: znad046_Supplementary_Data [file znad046_supplementary_data.docx]

**A prospective cohort study of operative outcomes in laparoscopic cholecystectomy using operative difficulty grade adjusted CUSUM analysis**

Isaac Tranter-Entwistle MBChB^1^

Corin Simcock^1^

Tim Eglinton MBChB, MMedSc, FRACS^1,2^

Saxon Connor MBChB, FRACS^2^

**Institutions:**

1. Department of Surgery, The University of Otago Medical School, Christchurch
2. Department of General Surgery Christchurch Hospital, CDHB

**Corresponding author.** Isaac Tranter-Entwistle, Department of Surgery, 36 Cashel Street, Christchurch Central, Christchurch 8013

**Supplementary Materials - Index**

| **Supplementary Methods** |  |
| --- | --- |
| Methodology for generating RA-CUSUM chart | *pag. 3-5* |
| **Supplementary Appendices**  Appendix S1: TRIPOD Statement | Pag.6-7 |
| **Supplementary Figures and Tables** |  |
| Table S1: Preoperative patient factors and investigations  Table S2: Intraoperative processes stratified by  operative grade  Table S3: Post-operative patient outcome stratified by operative grade | *pag. 8*  pag.9  Pag. 10 |
|  |  |
|  |  |

**Supplementary Methods**

**Methodology for generating RA-CUSUM chart**

The RA-CUSUM charts were generated using the methodology described by Steiner *et al^11^.*

A logistic regression model was generated to describe patient risk. For the Clavien-Dindo grade 3-5 (CD) complications, data from 2015 to the start of 2018 was used with operative grade (as designated by the north shore grading system^14,15^) as an explanatory variable.

$$\mathrm{logit}\left( p_{t} \right)=-3.83+0.56X_{t}$$

From this, individual operation scores (W_t_) were generated, based on operation outcome, difficulty, and odds ratios.

$$W_{t}=\left\{ \begin{aligned} log[\frac{\left( 1-p_{t}+R_{0}p_{t} \right)R_{A}}{\left( 1-p_{t}+R_{A}p_{t} \right)R_{0}}], &if y_{t}=1 \\ \log\left[ \frac{1-p_{t}+R_{0}p_{t}}{1-p_{t}+R_{A}p_{t}} \right], &if y_{t}=0 \end{aligned} \right.$$

The odds ratios R_0_ and R_A_ are the odds ratio under the null and alternate hypotheses, respectively. As the generated patient risk is based on the current conditions, R_0_ was set to 1. R_A_ can be set according to desirable and undesirable outcomes. For CD3-5 complications, an occurrence rate of 2% is deemed acceptable, while 3% is unacceptable^20,21^. This generated a value for R_A_ of 1.5 meaning the CUSUM process will detect 1.5 times increase in the odds of a CD3-5 complication. This individual outcome score is then used to generate a cumulative sum (X_t_) via:

$$X_{t}=\max\left( 0, X_{t-1}+W_{t} \right), X_{0}=0$$

This generation of CUSUM via the max function better allows the procedure to detect increases in the odds of an adverse outcome. This is because it prevents the accumulation of successful outcomes masking a run of adverse outcomes.

This also allows the use of a second CUSUM procedure designed to detect an improvement in performance. For the second CUSUM procedure Z_t_ is plotted, where Z_t_ is given by:

$$Z_{t}=\min\left( 0, Z_{t-1}-W_{t} \right), Z_{0}=0$$

For this an R_A_ of 2/3 was chosen. This represents a 50% improvement in the odds of an adverse outcome.

An important part of the generation of a RA-CUSUM is the setting of a control limit. If the CUSUM surpasses this limit, then the procedure has signaled. This means that the parameters of the measured process are likely to have changed – for the CUSUM with an R_A_ of 1.5, it indicates that the odds of an adverse outcome have increased past the acceptable level. For the procedure designed to detect improvement in performance, a signal from the CUSUM also indicates that the chart should be reformulated.

The choice of control limit for the CUSUM is important. A lower control limit will make the procedure more sensitive, but also more likely to signal ‘false positives’, where runs in bad performance may be due to random chance as opposed to a change in the odds ratio.

The sensitivity of a control limit can be quantified by an average run length (ARL), defined by the average amount of time in which a chart is expected to signal a false positive by chance. This can be approximated via several methods including with integral equations or with Markov Chains^12^. These two approaches are functionally similar, but Markov Chains are more general purpose, while integral equations and the approach to solve them can vary based on the CUSUM procedure^13^. This means Markov Chains are better suited to the current purpose of creating a framework that can adaptively generate CUSUM charts without field-specific expertise.

To implement the Markov Chain approach, the continuous CUSUM chart must be discretized into g+1 states, where g is the control limit multiplied by the scale. The transition probabilities for this set of states can then be formed into an *g+1* by *g+1* transition probability matrix, *Q*. At each position *i,j* in matrix Q, q_i,j_ is the probability of moving from state *i* to state *j.* This is the probability of the next patient having a score of *j-i.*

State g indicates the absorbing state. Reaching it represents the procedure signaling, and as the transition probability matrix is generated based on current odds ratios, this signal would be a false alarm. The characteristics of Markov Chains can be used to identify the average number of runs it takes to get from state 0 to the absorbing state.

First, the fundamental matrix is generated:

$$F=\left( I-Q \right)^{-1}$$

Where *I* is the g by g identity matrix and *Q* is the transition probability matrix with the row and column representing the absorbing state removed.

The *i, j*-th entry of the fundamental matrix conveys the average number of times the process is in the state *j* prior to absorption, given that it began in the state *i*. If **1** is a g by 1 column vector of ones*,* $F*\boldsymbol{1}$ gives a g by 1 vector containing the ARL before the process signals, starting in every discretized starting state.

The ability to find the ARL of a procedure makes it possible to set a control limit *h* for any desired sensitivity. In the context of CD3-5 complications an ARL of 4500 was targeted. At the current rate of operations, this means a false signal from the CUSUM procedure is expected every 8-9 years. In the procedure for detecting deteriorations, a control limit of 2.5 was used, and for improvements a control limit of -8 was used. The outcomes are shown in figure 2.

**Supplementary Appendix**

| **Section/Topic** | **Item** | **Checklist Item** | **Page** |
| --- | --- | --- | --- |
| **Title and abstract** | | | |
| Title | 1 | Identify the study as developing and/or validating a multivariable prediction model, the target population, and the outcome to be predicted. | Yes |
| Abstract | 2 | Provide a summary of objectives, study design, setting, participants, sample size, predictors, outcome, statistical analysis, results, and conclusions. | NA -  Short Report |
| **Introduction** | | | |
| Background and objectives | 3a | Explain the medical context (including whether diagnostic or prognostic) and rationale for developing or validating the multivariable prediction model, including references to existing models. | Para 1 |
|  | 3b | Specify the objectives, including whether the study describes the development or validation of the model or both. | Para 1 |
| **Methods** | | | |
| Source of data | 4a | Describe the study design or source of data (e.g., randomized trial, cohort, or registry data), separately for the development and validation data sets, if applicable. | Para 2 |
|  | 4b | Specify the key study dates, including start of accrual; end of accrual; and, if applicable, end of follow-up. | Para 4 |
| Participants | 5a | Specify key elements of the study setting (e.g., primary care, secondary care, general population) including number and location of centres. | Para 2 |
|  | 5b | Describe eligibility criteria for participants. | Para 4 |
|  | 5c | Give details of treatments received, if relevant. | Para 4 |
| Outcome | 6a | Clearly define the outcome that is predicted by the prediction model, including how and when assessed. | Para 5 |
|  | 6b | Report any actions to blind assessment of the outcome to be predicted. | NA |
| Predictors | 7a | Clearly define all predictors used in developing or validating the multivariable prediction model, including how and when they were measured. | Para 5 |
|  | 7b | Report any actions to blind assessment of predictors for the outcome and other predictors. | NA |
| Sample size | 8 | Explain how the study size was arrived at. | NA |
| Missing data | 9 | Describe how missing data were handled (e.g., complete-case analysis, single imputation, multiple imputation) with details of any imputation method. | Appendix 1 |
| Statistical analysis methods | 10a | Describe how predictors were handled in the analyses. | Appendix 1 |
|  | 10b | Specify type of model, all model-building procedures (including any predictor selection), and method for internal validation. | Appendix 1 |
|  | 10d | Specify all measures used to assess model performance and, if relevant, to compare multiple models. | Appendix 1 |
| Risk groups | 11 | Provide details on how risk groups were created, if done. | Appendix 1 |
| **Results** | | | |
| Participants | 13a | Describe the flow of participants through the study, including the number of participants with and without the outcome and, if applicable, a summary of the follow-up time. A diagram may be helpful. | Appendix 2 |
|  | 13b | Describe the characteristics of the participants (basic demographics, clinical features, available predictors), including the number of participants with missing data for predictors and outcome. | Appendix 2 |
| Model development | 14a | Specify the number of participants and outcome events in each analysis. | Para 7 |
|  | 14b | If done, report the unadjusted association between each candidate predictor and outcome. | Appendix 1 |
| Model specification | 15a | Present the full prediction model to allow predictions for individuals (i.e., all regression coefficients, and model intercept or baseline survival at a given time point). | Appendix 1 |
|  | 15b | Explain how to the use the prediction model. | Appendix 1 / Para 9 |
| Model performance | 16 | Report performance measures (with CIs) for the prediction model. | NA |
| **Discussion** | | | |
| Limitations | 18 | Discuss any limitations of the study (such as nonrepresentative sample, few events per predictor, missing data). | Para 9 |
| Interpretation | 19b | Give an overall interpretation of the results, considering objectives, limitations, and results from similar studies, and other relevant evidence. | Para 8 |
| Implications | 20 | Discuss the potential clinical use of the model and implications for future research. | Para 9 |
| **Other information** | | | |
| Supplementary information | 21 | Provide information about the availability of supplementary resources, such as study protocol, Web calculator, and data sets. | Y |
| Funding | 22 | Give the source of funding and the role of the funders for the present study. | Y |

**Supplementary Figures and Tables**

**Table S1: Preoperative patient factors and investigations**

| Factor | All Patients  n = 4663 | Grade 1 (%)  1737(37.3) | Grade 2 (%)  1755 (37.6) | Grade 3 (%)  365 (7.8) | Grade 4 (%)  382 (8.2) |
| --- | --- | --- | --- | --- | --- |
| Admission  Acute  Elective | 2837 (61)  1826 (39) | 756 (43)  981 (57) | 1297 (74)  458 (26) | 224 (61)  141 (39) | 373 (98)  9 2 (2) |
| Gender  Female  Male | 3122 (67)  1541 (33) | 1328 (77)  409 (23) | 1153 (66)  602 (34) | 195 (53)  170 (47) | 150 (39)  232 (61) |
| Age in years (IQR) | 53 (38-67) | 49 (34-65) | 53 (38-66) | 61 (47-72) | 63 (53-73) |
| Indication  Cholecystitis  Acalculous Cholecystitis  Chronic Cholecystitis  Biliary Pain  Cholangitis  Fistula  Choledocholithiasis  Pancreatitis  Gallbladder Polyp  Missing | 1271 (27)  8 (0)  216 (5)  927 (20)  119 (0)  15 (0)  254 (5)  436 (9)  77 (2)  1340 (29) | 84 (5)  2 (0)  27 (2)  671 (39)  27 (2)  2 (0)  137 (8)  268 (15)  62 (3)  457 (26) | 754 (43)  2 (0)  132 (8)  146 (8)  56 (3)  5 (0)  77 (4)  121 (7)  6 (0)  456 (26) | 124 (34)  1 (0)  36 (10)  35 (10)  16 (4)  6 (2)  29 (8)  26 (7)  2 (1)  90 (25) | 260 (68)  3 (1)  6 (2)  0 (0)  13 (3)  2 (1)  4 (1)  4 (1)  1 (0)  89 (32) |
| Bilirubin  Normal  Elevated  Missing | 3653 (78)  691 (15)  319 (7) | 1485 (85)  183 (11)  69 (4) | 1396 (80)  299 (17)  42 (2) | 291 (80)  56 (15)  18 (5) | 252 (65)  121 (32)  9 (2) |

| Factor | All Patients  n=4663 | Grade 1 (%) | Grade 2 (%) | Grade 3 (%) | Grade 4 (%) |
| --- | --- | --- | --- | --- | --- |
| Procedure  Laparoscopic Cholecystectomy  Laparoscopic Cholecystectomy + IOC  LCBDE – Main Duct  LCBDE – Transcystic  Open CBDE  Open Cholecystectomy  Combined Procedure  Radical Cholecystectomy | 3325 (71)  1121 (24)  28 (1)  54 (1.2)  8 (0)  88 (2)  25 (1)  10 (0) | 1221 (70)  467 (27)  5 (0)  23 (1)  0 (0)  11 (1)  8 (1)  2 (0) | 1247 (71)  444 (25)  14 (1)  18 (1)  5 (0)  22 (1)  3 (0)  2 (0) | 264 (76)  74 (20)  5 (1)  10 (3)  0 (0)  10 (3)  2 (1)  0 (0) | 289 (76)  64 (17)  3 (1)  2 (1)  0 (0)  19 (5)  3 (0)  0 (0) |
| Role  Trainee  Consultant | 2523 (54)  2140 (46) | 1061 (61)  676 (39) | 944 (54)  811 (46) | 156 (43)  209 (57) | 204 (53)  178 (46) |
| Gallbladder Reflected Cephalad  Not possible/difficult  Required dissection of viscera/adhesions  Yes  Missing | 54 (1)  589(13)  3821 (82)  199 (4) | 11 (1)  131 (8)  1576(91)  19 (1) | 20 (1)  234 (13)  1736 (85)  19 (1) | 11 (3)  93 (26)  256 (70)  5 (1) | 9 (2)  111 (29)  253 (66)  9 (2) |
| Rouviere’s Sulcus Identified  Yes  No  Missing | 3915 (84)  518 (11)  230 (5) | 1565 (90)  149(9)  23 (1) | 1507 (86)  214(12)  34 (2) | 296 (81)  63(17)  6 (2) | 301 (79)  65(17)  16 (4) |
| Anterior and posterior peritoneum released  Diathermy  Dissection  Missing | 2273 (49)  2125 (46)  265 (6) | 992 (57)  720 (42)  25 (1) | 848 (48)  861 (49)  46 (3) | 159 (44)  182 (50)  24 (6) | 131 (34)  229 (60)  22 (6) |
| Calot’s Triangle Displayed  Diathermy  Dissection  Unable to be achieved  Missing | 388 (8)  3743 (80)  314 (7)  218 (5) | 182 (11)  1532 (88)  5 (0)  18 (1) | 153 (9)  1444 (82)  125 (7)  33 (2) | 20 (6)  242 (66)  96 (26)  7 (2) | 10 (3)  275 (72)  84 (22)  13 (3) |
| CVS Achieved  Yes  No  Missing | 4131(88)  314 (7)  218 (5) | 1714 (99)  5 (0)  18 (0) | 1597 (91)  125 (7)  33 (2) | 262 (72)  96 (26)  7 (2) | 285 (75)  84 (22)  13 (3) |
| Converted to Open  Yes  No | 46 (1)  4617 (99) | 15 (2)  831 (98) | 17 (2)  858 (98) | 6 (3)  191 (97) | 7 (3)  204 (97) |
| Operative time in minutes (IQR) | 83 (65 – 108) | 72 (57-88) | 89 (69-113) | 99 (77-128) | 102 (83-126) |

**Table S2: Intraoperative processes stratified by operative grade**

Table S3: Post-operative patient outcome stratified by operative grade

| Factor | All patients  n = 4663 | Grade 1 | Grade 2 | Grade 3 | Grade 4 |
| --- | --- | --- | --- | --- | --- |
| Clavien Dindo Grade 3-5 Complication  Yes  No | 100 (2)  4563 (98) | 27 (2)  1710 (98) | 27 (2)  1728 (98) | 12 (3)  353 (97) | 18 (5)  364 (95) |
| Clavien Dindo  3  4  5 | 94 (2)  3 (0)  3 (0) | 26  2  0 | 27  0  0 | 10  1  1 | 17  0  1 |
| Bile Leak  Yes  No | 35 (1)  4628 (99) | 7 (0.4)  1730 (99.6) | 5 (0.3)  1750 (99.7) | 7 (2)  358 (98) | 11 (3)  371 (97) |
| Length of Stay in Days (IQR) | 2 (1-4) | 1 (1-4) | 3 (1-4) | 3 (1-5) | 4 (3-5) |
